# Single channel kinetic analysis of the cAMP effect on *I_Ks_* mutants, S209F and S27D/S92D

**DOI:** 10.1080/19336950.2018.1499369

**Published:** 2018-08-28

**Authors:** Emely Thompson, Jodene Eldstrom, David Fedida

**Affiliations:** Department of Anesthesiology, Pharmacology and Therapeutics, University of British Columbia, Vancouver, BC, Canada

**Keywords:** cAMP, *I_Ks_*, KCNE1, KCNQ1 S27D/S92D, KCNQ1 S209F

## Abstract

The *I_Ks_* current is important in the heart’s response to sympathetic stimulation. *β*-adrenergic stimulation increases the amount of *I_Ks_* and creates a repolarization reserve that shortens the cardiac action potential duration. We have recently shown that 8-CPT-cAMP, a membrane-permeable cAMP analog, changes the channel kinetics and causes it to open more quickly and more often, as well as to higher subconductance levels, which produces an increase in the *I_Ks_* current. The mechanism proposed to underlie these kinetic changes is increased activation of the voltage sensors. The present study extends our previous work and shows detailed subconductance analysis of the effects of 8-CPT-cAMP on an enhanced gating mutant (S209F) and on a double pseudo-phosphorylated *I_Ks_* channel (S27D/S92D). 8-CPT-cAMP still produced kinetic changes in S209F + KCNE1, further enhancing gating, while S27D/S92D + KCNE1 showed no significant response to 8-CPT-cAMP, suggesting that these last two mutations fully recapitulate the effect of channel phosphorylation by cAMP.

## Introduction

The *I_Ks_* potassium current is involved in terminating the plateau phase of the cardiac action potential and becomes more important as sympathetic activity increases the heart rate [1]. Under sympathetic stimulation, the *I_Ks_* current is enhanced in a cAMP-dependent fashion, leading to faster action potential repolarization, which shortens the action potential and allows adequate time for ventricular refilling [1–4]. Mutations in the channel can give rise to long and short QT syndrome as well as familial atrial fibrillation [5–7].

A homotetramer of peptides encoded by the *KCNQ1* gene (Q1) form the channel and are modulated by KCNE1 (E1) accessory subunits to produce the *I_Ks_* current. When E1 subunits are a part of the channel complex, the current is converted from one that activates rapidly and inactivates, to one with very slow activation, deactivation kinetics and no inactivation [8,9]. The number of these accessory subunits in the channel complex can vary between 1 to 4 units per channel [10], which allows the channel to be modulated and perhaps regulated by the number of E1s in the complex. Two residues in the N-terminus are phosphorylated by cAMP, S27 and S92 [11–13] and under sympathetic stimulation, the N-terminus of Q1 becomes phosphorylated by protein kinase A (PKA), which is part of a macromolecular complex bound to the C-terminal domain of Q1 [14].

Exogenous 8-CPT-cAMP decreases the voltage threshold for activation and increases the magnitude of *I_Ks_*, but only when the KCNE1 subunit and yotiao are present [1,13,15–18]. Single channel recording has revealed the detailed changes in channel kinetics in the presence of 8-CPT-cAMP. We showed that the increase in current observed upon 8-CPT-cAMP addition is caused by channels opening more quickly, more often and to higher open sublevels, and suggested that these effects were caused by increased activation of the voltage sensor domains (VSDs) [15]. Mutant Q1 channels with enhanced or fully activated VS were used to characterize this effect.

In this addendum, we have further analysed the single channel kinetics of two Q1 mutants used in the original paper. One is S209F, a high open probability (Po) mutant with enhanced gating, where 8-CPT-cAMP has some effect on the subconductance occupancy. The other is a double phosphomimetic mutant (S27D/S92D), which appeared to show no response to 8-CPT-cAMP, but that has kinetic properties more similar to wild-type than the single phosphomimetic mutant, S27D.

## Results and discussion

### Kinetic analysis of 8-CPT-cAMP on an enhanced gating mutant, S209F

S209F is a kinetic gain-of-function mutation, which can be described as having a preferentially activated VSD stabilizing the open state. When complexed with E1, it has a Po between 0.6 and 0.7 [15,19], which is about 4x that of wild-type Q1 + E1 (0.15–0.2) [10,19,20]. In our previous paper [15], at the single channel level, there was no change in the Po (control 0.71 and 0.74 in 8-CPT-cAMP at + 60 mV [15]), but there was a reduction in the number of closed events and some changes in subconductance levels from the all-point histogram distribution in the presence of 8-CPT-cAMP.

**Figure 1.**
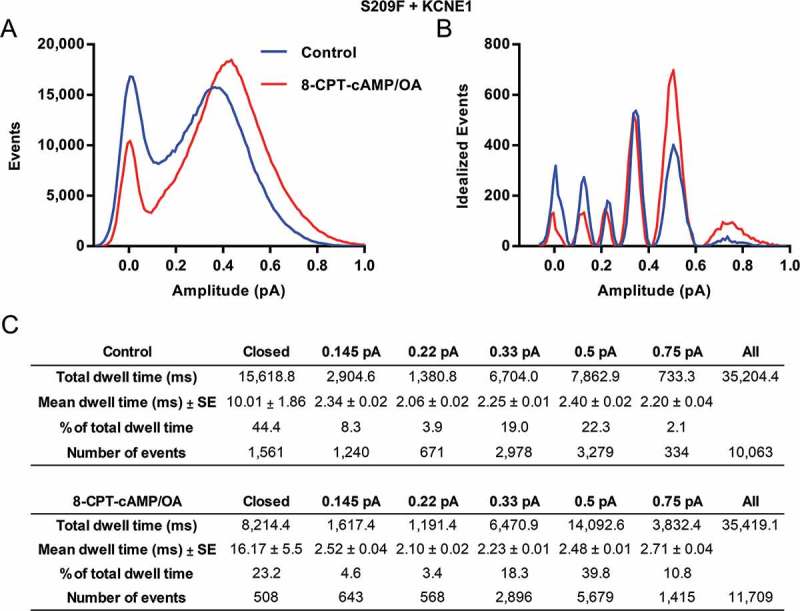
**Subconductance analysis of Q1 S209F + E1 before and after 8-CPT-cAMP/Okadaic acid (OA)**. (a) Raw all-points histograms display the distribution of amplitudes from 20 active control sweeps (blue) and 20 sweeps in the presence of 200 µM 8-CPT-cAMP and 0.2 µM OA (red) from one representative cell were pulsed to + 60 mV for 4 s from a holding potential of −80 mV and filtered at 500 Hz. (b) Five initial thresholds used for idealization were 0.145, 0.22, 0.33, 0.5 and 0.75 pA and are shown in the table headers. The final idealization levels for control histograms were 0.13 ± 0.03, 0.23 ± 0.03, 0.35 ± 0.03, 0.50 ± 0.05 and 0.70 ± 0.10. In the presence of 8-CPT-cAMP/OA the levels were, 0.13 ± 0.03, 0.23 ± 0.03, 0.35 ± 0.03, 0.51 ± 0.05, 0.71 ± 0.07. (c) Total and mean dwell times (milliseconds) for each of the different thresholds, the percentage of time spent at each level, and the number of events at each threshold before and after 8-CPT-cAMP. These data were filtered at 0.5 kHz, and the bin width used was 0.01 pA. Only events longer than 1.5 ms were included. One-way ANOVA was used to compare control and 8-CPT-cAMP mean dwell times of each sublevel. The mean dwell times between control and 8-CPT-cAMP were significantly different (p-value < 0.0001). The p-values for all other sublevels were > 0.9998.

To further investigate S209F changes in subconductance in response to 8-CPT-cAMP, analysis of 20 S209F + E1 sweeps before and after 8-CPT-cAMP was carried out, and shows changes in the substate occupancy levels in the presence of 8-CPT-cAMP (Figure 1). The closed dwell time percentage in control is ~ 44%, which is considerably less than EQ even in the presence of 8-CPT-cAMP (Control 88% and 8-CPT-cAMP 79%, Thompson et al [15]. This closed dwell time drops to ~ 21% in the presence of 8-CPT-cAMP. The mean closed dwell time duration are also significantly shorter in the presence of 8-CPT-cAMP (p-value: < 0.0001). The two highest sublevels 0.5 and 0.75 pA both increased in total dwell time by ~ 18 and 9% respectively (Figure 1(c)), showing a shift in occupancy to higher sublevels. There are no significant changes in the mean dwell times at any of the open levels (p-value: > 0.999) (Figure 1(c)).

The S209 residue is buried in the hydrophobic core in the open state. The mutation converts a polar uncharged residue into a bulky hydrophobic one, which is thought to stabilize the open state and destabilize the closed state, resulting in gain-of-function activity [21]. This mutant can presumably gate normally as the voltage sensor is not “locked” unlike the E160R/R237E (E1R/R4E) mutant, which is locked in an activated state and appears to be unaffected by 8-CPT-cAMP [14]. While S209F has been described as “fully” activated, 8-CPT-cAMP does appear to further stabilize the open state by increasing events at higher subconducting states (Figure 1). A reduction to ~ 20% in closed time in the presence of 8-CPT-cAMP may also suggest a destabilization of the closed state (Figure 1(c)).

**Figure 2.**
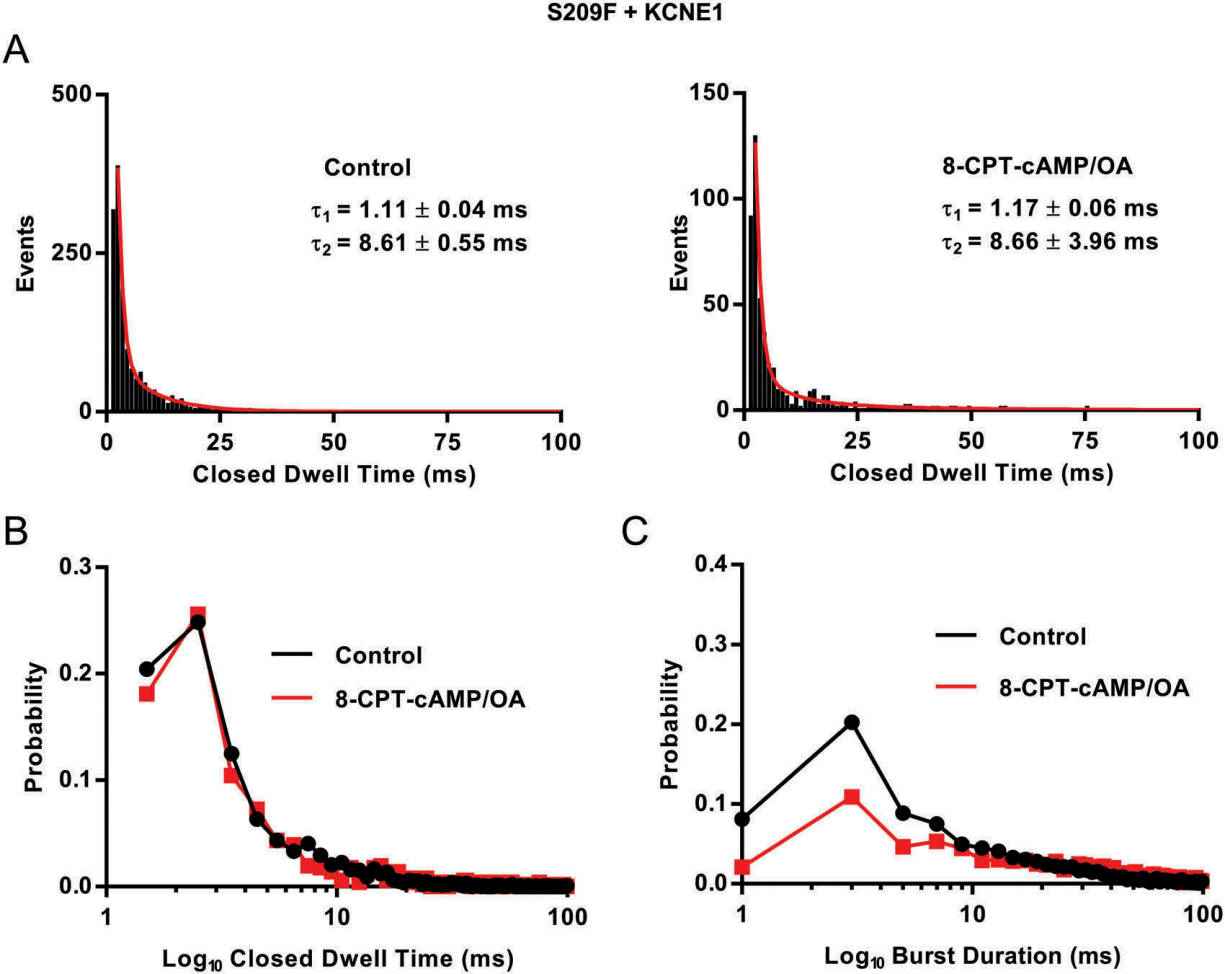
**Q1 S209F + E1 closed dwell times and burst analysis**. (a) Closed dwell time distributions for S209F + E1 from 20 sweeps before and 20 sweeps after 200 µM 8-CPT-cAMP/0.2 µM OA  from one representative cell. Data were fitted with the sum of two exponential functions. τ1: 1.11 ± 0.04 ms (area under the curve, AUC, 309 ± 5.5) and τ2: 8.61 ± 0.55 ms (AUC 75.4 ± 5.0 in control. After 8-CPT-cAMP/OA, τ1: 1.17 ± 0.06 ms (AUC 110 ± 3.9) and τ2: 8.66 ± 3.96 ms (AUC 14.2 ± 3.0). Bin width was 1 ms. (b) Probability distribution of closed time durations in control (black) and after 8-CPT-cAMP/OA (red), from data in A. (c) Probability distributions of burst durations based on open dwell time histograms, control (black) and after 8-CPT-cAMP/OA (red). Dwell-time histograms were fitted with the sum of three exponential functions in control τ1: 1.70 ± 0.09 ms (AUC 334 ± 10.7), τ2: 13.87 ± 2.41 ms (AUC 140 ± 16.7) and τ3: 44.38 ± 12.75 ms (AUC 39.5 ± 21.6). In 8-CPT-cAMP only the sum of two exponential functions could be fit, τ1: 2.62 ± 0.29 ms (AUC 88.2 ± 4.5) and τ2: 41.35 ± 3.36 ms (AUC 51.5 ± 2.7). Bin width was 2 ms. Only events longer than 1.5 ms in duration were used in this analysis.

The closed dwell time constants between control and 8-CPT-cAMP remain relatively unaffected by cAMP (control τ_1_: 1.11 ± 0.04, τ_2_: 8.61 ± 0.55 and 8-CPT-cAMP τ_1_: 1.17 ± 0.06, τ_2_: 8.66 ± 3.96 ms) (Figure 2(a)) and are similar to the closed times of EQ in the presence of 8-CPT-cAMP [15]. The probability distribution of the closed dwell times shows that the closed times are not affected by 8-CPT-cAMP (Figure 2(b)).

Data in Figure 2(c) show the probability distribution plot of burst durations before and after 8-CPT-cAMP. There is a small decrease in the number of short and intermediate bursts (Figure 2(c)). The open dwell times in control could be fit with three time constants (τ_1_: 1.70 ± 0.09, τ_2_: 13.87 ± 2.41, τ_3_: 44.38 ± 12.75 ms), however the second time constant was absent in the presence of 8-CPT-cAMP, as only two time constants could be fit (τ_1_: 2.62 ± 0.29 and τ_2_: 41.35 ± 3.36 ms). 8-CPT-cAMP may be having a small effect on the pore as well as the VSD. However, the fits for these 8-CPT-cAMP data may reflect the presence of fewer events. Comparing the EQ burst probability distribution plots [15] to the S209F + E1 plots, S209F has a wider range of burst durations, whereas EQ has the majority of its events taking place at the shorter burst durations.

S209F + E1 is still able to respond to 8-CPT-cAMP at the single channel level, with a reduction in the total closed dwell time and an increase in the time spent at the higher subconducting levels. This suggests that even though this channel has enhanced activation, it can be still further increased by cAMP.

### Single channel analysis of Q1 double-phosphomimetic mutant S27D/S92D

The S27 residue in the N-terminus of Q1 is an important phosphorylation site during adrenergic stimulation [13], which can be pseudo-phosphorylated by mutating the serine to aspartic acid, S27D [22]. This mutation produces currents of greater magnitude and with a more hyperpolarized V_1/2_ [22], but can be further phosphorylated, which suggests that there are other potential significant phosphorylation residues [15]. S92 has been shown by others to be phosphorylated [11], so we investigated the importance of this residue by using a double phosphomimetic mutant (S27D/S92D) and tested its response to 8-CPT-cAMP.

**Figure 3.**
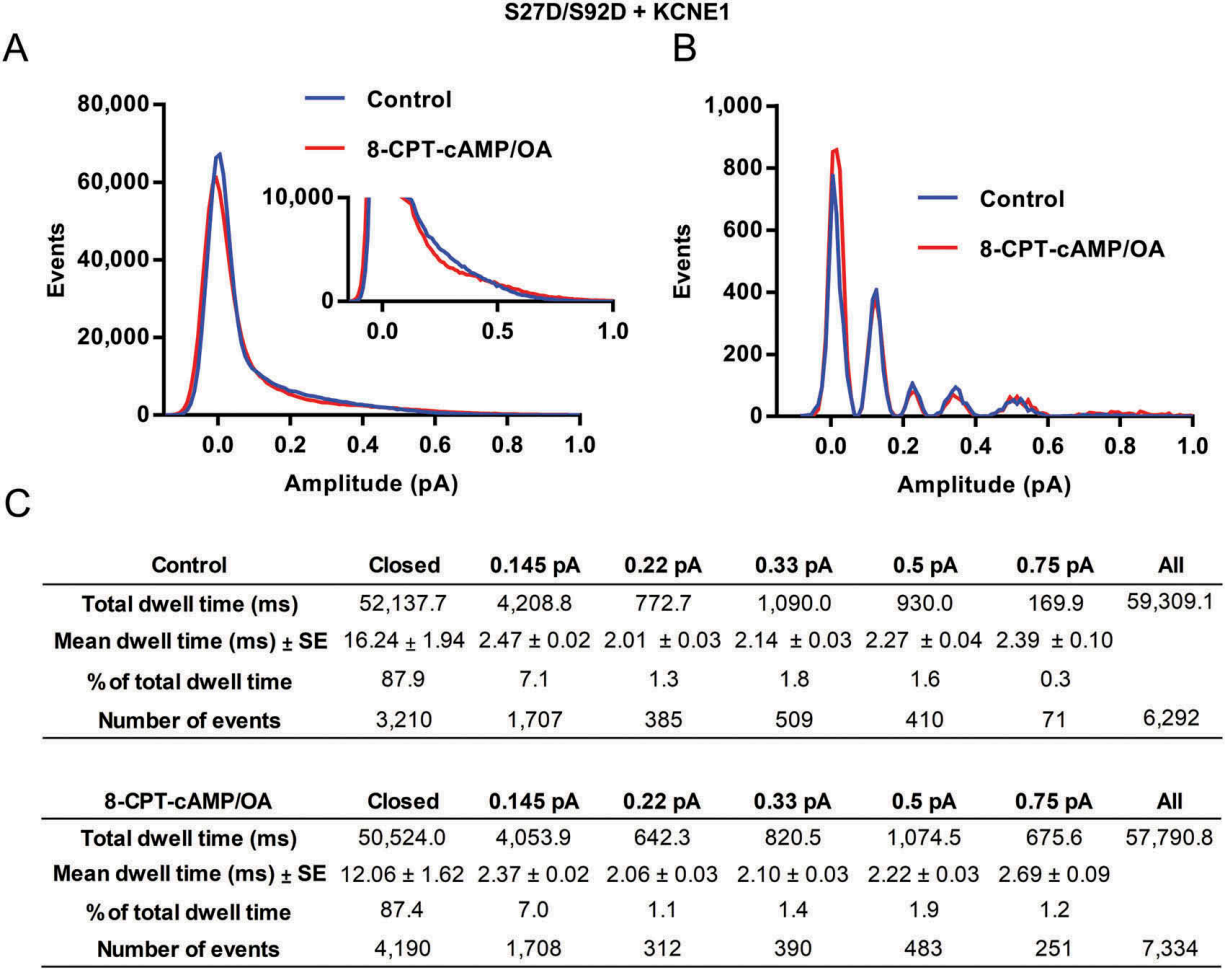
**Subconductance analysis of Q1 S27D/S92D + E1 before and after 8-CPT-cAMP/OA**. (a) Raw all-points histograms display the distribution of amplitudes from 20 active control sweeps (blue) and 20 sweeps in the presence of 200 µM 8-CPT-cAMP and 0.2 µM OA (red) (20 sweeps before and after 8-CPT-cAMP from one representative cell). (b) Five initial thresholds used for idealization were 0.145, 0.22, 0.33, 0.5 and 0.75 pA and are shown in the table headers. The final idealization levels for control histograms were 0.12 ± 0.03, 0.23 ± 0.03, 0.34 ± 0.04, 0.50 ± 0.05 and 0.71 ± 0.07. In the presence of 8-CPT-cAMP/OA the levels were, 0.11 ± 0.03, 0.23 ± 0.03, 0.34 ± 0.04, 0.51 ± 0.05, 0.74 ± 0.10. (c) Total and mean dwell times (milliseconds) for each of the different thresholds, the percentage of time spent at each level, and the number of events at each threshold before and after 8-CPT-cAMP. These data were filtered at 0.5 kHz, and the bin width used was 0.01 pA. Only events longer than 1.5 ms were included. A one-way ANOVA was used to compare control and 8-CPT-cAMP mean dwell times of each sublevel. None of the 8-CPT-cAMP mean dwell times were significantly different from control (p-value between closed sublevels was 0.1355, all other sublevels were > 0.9999).

At the whole-cell level, there was no change in current size or V_1/2_ of activation in response to 8-CPT-cAMP. At the single channel level, there was also no change in first latency or any significant change in the all-points histograms (Figure 3(a) & [15]). We carried out further investigation by analyzing subconductance occupancy (Figure 3). The idealized histograms in Figure 3(b) show no significant change in the substate occupancy rates in the presence of 8-CPT-cAMP and no significant change in the time spent at each subconducting level between control and 8-CPT-cAMP (Figure 3(c)) (p-value: > 0.1).

**Figure 4.**
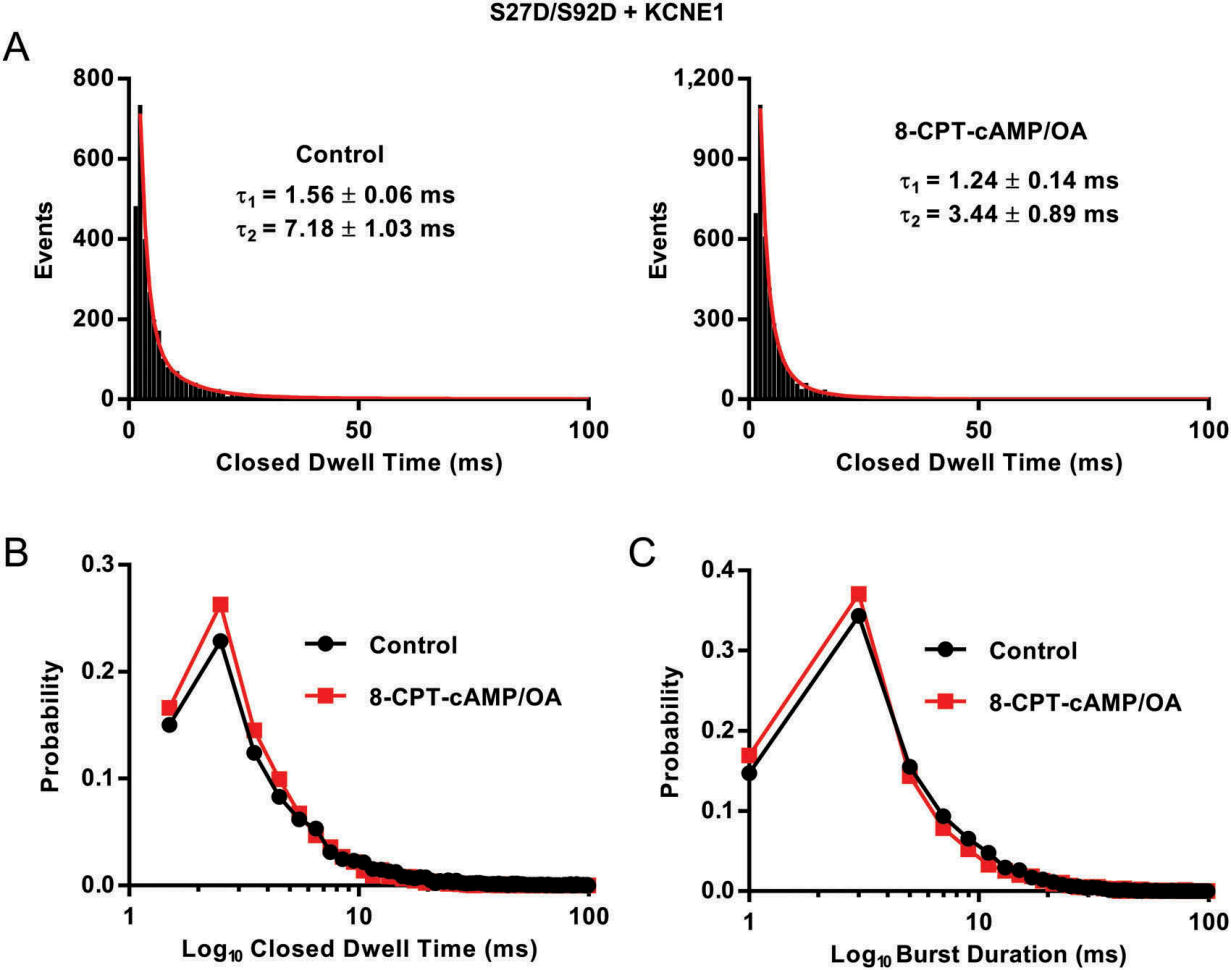
**Q1 S27D/S92D + E1 closed dwell times and burst analysis**. (a).Closed dwell time distributions for S27D/S92D + E1 from 20 sweeps before and 20 sweeps after 200 µM 8-CPT-cAMP/0.2 µM OA (20 sweeps before and after 8-CPT-cAMP from one representative cell). Data were fitted with the sum of two exponential functions. τ1: 1.56 ± 0.06 ms (AUC 547 ± 20.3) and τ2: 7.18 ± 1.03 ms (AUC 158 ± 16.4) in control. After 8-CPT-cAMP/OA, τ1: 1.24 ± 0.14 ms (AUC 616 ± 132.3) and τ2: 3.44 ± 0.89 ms (AUC 416 ± 97.6). Bin width was 1 ms. (b) Probability distribution of closed time durations in control (black) and after 8-CPT-cAMP/OA (red), from data in A. (c) Probability distributions of burst durations based on open dwell time histograms, control (black) and after 8-CPT-cAMP/OA (red). Dwell-time histograms were fitted with the sum of three exponential functions in control τ1: 1.11 ± 0.12 ms (AUC 464 ± 56.8), τ2: 4.50 ± 0.88 ms (AUC 469 ± 56.9) and τ3: 11.10 ± 2.99 ms (AUC 126 ± 92.4). In 8-CPT-cAMP only the sum of two exponential functions could be fit, τ1: 1.59 ± 0.06 ms (AUC 792 ± 28.3) and τ2: 7.82 ± 1.52 ms (AUC 229 ± 20.1). Bin width was 2 ms. Only events longer than 1.5 ms in duration were used in this analysis.

With respect to the closed and open dwell times (Figure 4), two resolvable closed states were found for both control and 8-CPT-cAMP events. The faster time constant in control and cAMP was similar, 1.56 ± 0.06 and 1.24 ± 0.14 ms respectively (Figure 4(a)). The second time constant was slower in control 7.18 ± 1.03 compared to 3.44 ± 0.89 ms in 8-CPT-cAMP (Figure 4(a)). However, this could be due to a smaller number of events in the control fit. When the closed dwell times are plotted as a probability distribution (Figure 4(b)), there is no clear effect of 8-CPT-cAMP on the closed dwell times. Finally, 8-CPT-cAMP does not appear to affect the open dwell time distribution (Figure 4(c)).

In *Xenopus* oocytes, the Q1 S27D/S92D mutant appears to have a more hyperpolarized V_1/2_ compared to wild-type [23], which is what one might expect of a phosphomimetic mutant. However, in mammalian cells this construct produced a V_1/2_ that was more depolarized than wild-type (Q1 S27D/S92D + E1 V_1/2_: 47.5 ± 8.6 mV and Q1 + E1 V_1/2_: 25.1 ± 2.5 mV [15]). This was surprising, and interestingly from the single channel analysis (Figure 4), we can see that this mutant does not quite behave like wild-type either. There is no visible main active opening amplitude peak of ~ 0.45 pA that is usually observed in wild-type channels (Figure 3(a)), although the first latency of the channel was similar to that of EQ (1.62 ± 0.08 s and 1.61 ± 0.13 s respectively) [15]. While this double pseudo-phosphorylation mutant does not appear to be affected kinetically by 8-CPT-cAMP, this mutant still showed altered single channel kinetics compared to wild-type.

## Conclusion

Subconductance analysis allows us to better understand the kinetic changes that 8-CPT-cAMP causes at a single channel level. Even though S209F has highly enhanced gating, 8-CPT-cAMP is able to stabilize the channel open state further. The double pseudo phosphorylated mutant, S27D/S92D does not respond to 8-CPT-cAMP.

## Methods

### Reagents

To activate PKA, 200 μM of 8-(4-chlorophenylthio)adenosine 3′,5′-cyclic monophosphate sodium salt (8-CPT-cAMP) (Sigma-Aldrich), a membrane-permeable cAMP analog, was used. To sustain the cAMP analog, an inhibitor of protein phosphatase 1 (PP1) Okadaic acid (OA) (EMD Millipore) was used at 0.2 µM concentration.

### Molecular biology

Constructs used were as previously described in Thompson et al. [15].

### Cell culture and transfections

For single channel recording, *ltk^−^* mouse fibroblast (LM) cells were used. They were grown in Minimum Eagle Medium (Thermo Fisher Scientific) with 10% fetal bovine serum, 100 U/ml penicillin and 100 µg/ml streptomycin (Thermo Fisher Scientific) added. Cells were kept at 37°C in a humid atmosphere containing 5% CO_2_. Constructs were overexpressed by transient transfection using Lipofectamine2000 (Thermo Fisher Scientific). Q1 S209F or S27D/S92D, E1-GFP and AKAP-9 (required cofactor for phosphorylation of Q1) were co-expressed in a 1:3:1 ratio. All recordings were performed 24–48 hr after transfection at room temperature.

### Patch-clamp electrophysiology

Single-channel recording methodology was performed as previously described in Thompson et al. and Werry et al. [15,19]. Data were obtained and analyzed using Axopatch hardware and pCLAMP 10.5 software (Molecular Devices).

### Solutions

The solutions used for both single-channel recordings were as described in Thompson et al [15].

## Data analysis

Data analysis was performed using Prism 7 (GraphPad Software). Single-channel data were acquired at 2 kHz and digitized using Digidata 1440A hardware (Molecular Devices). All recordings used for subconductance analysis were filtered at 500 Hz. Only events over 1.5 ms in duration were included in the analysis. One-way ANOVAs using Sidak’s multiple comparisons test were performed using Prism 7 (GraphPad Software).

### Statistics

Results shown here are mean values ± SE (Prism 7, GraphPad Software).

## References

[1] Terrenoire C, Clancy CE, Cormier JW, et al Autonomic control of cardiac action potentials. Circ Res. 2005;96:e25–e34. 15731462 10.1161/01.RES.0000160555.58046.9a

[2] Stengl M, Volders PGA, Thomsen MB, et al Accumulation of slowly activating delayed rectifier potassium current (I(Ks)) in canine ventricular myocytes. J Physiol. 2003;551:777–786. 12819301 10.1113/jphysiol.2003.044040 PMC2343293

[3] Jost N, Virág L, Bitay M, et al Restricting excessive cardiac action potential and QT prolongation. Circulation. 2005;112:1392–1399. 16129791 10.1161/CIRCULATIONAHA.105.550111

[4] Silva J, Rudy Y. Subunit interaction determines I(Ks) participation in cardiac repolarization and repolarization reserve. Circulation. 2005;112:1384–1391. 16129795 10.1161/CIRCULATIONAHA.105.543306 PMC1820744

[5] Moss AJ, Schwartz PJ, Crampton RS, et al The long QT syndrome. Prospective longitudinal study of 328 families. Circulation. 1991;84:1136–1144. 1884444 10.1161/01.cir.84.3.1136

[6] Chen YH, Xu SJ, Bendahhou S, et al KCNQ1 gain-of-function mutation in familial atrial fibrillation. Science. 2003;299:251–254. 12522251 10.1126/science.1077771

[7] Bellocq C, van Ginneken ACG, Bezzina CR, et al Mutation in the KCNQ1 gene leading to the short QT-interval syndrome. Circulation. 2004;109:2394–2397. 15159330 10.1161/01.CIR.0000130409.72142.FE

[8] Sanguinetti MC, Curran ME, Zou A, et al Coassembly of K(V)LQT1 and minK (IsK) proteins to form cardiac I(Ks) potassium channel. Nature. 1996;384:80–83. 8900283 10.1038/384080a0

[9] Barhanin J, Lesage F, Guillemare E, et al K(V)LQT1 and lsK (minK) proteins associate to form the I(Ks) cardiac potassium current. Nature. 1996;384:78–80. 8900282 10.1038/384078a0

[10] Murray CI, Westhoff M, Eldstrom J, et al Unnatural amino acid photo-crosslinking of the I(Ks) channel complex demonstrates a KCNE1: KCNQ1stoichiometry of up to 4:4. eLife. 2016;5:e11815. 26802629 10.7554/eLife.11815 PMC4807126

[11] Lundby A, Andersen MN, Steffensen AB, et al In vivo phosphoproteomics analysis reveals the cardiac targets of beta-adrenergic receptor signaling. Sci Signal. 2013;6:rs11. 23737553 10.1126/scisignal.2003506

[12] Lopes CM, Remon JI, Matavel A, et al Protein kinase A modulates PLC-dependent regulation and PIP2-sensitivity of K+ channels. Channels. 2007;1:124–134. 18690021 10.4161/chan.4322

[13] Marx SO, Kurokawa J, Reiken S, et al Requirement of a macromolecular signaling complex for beta adrenergic receptor modulation of the KCNQ1-KCNE1 potassium channel. Science. 2002;295:496–499. 11799244 10.1126/science.1066843

[14] Haitin Y, Wiener R, Shaham D, et al Intracellular domains interactions and gated motions of I(KS) potassium channel subunits. Embo J. 2009;28:1994–2005. 19521339 10.1038/emboj.2009.157 PMC2718281

[15] Thompson E, Eldstrom J, Westhoff M, et al cAMP-dependent regulation of *I_Ks_* single-channel kinetics. J Gen Physiol. 2017;149:781–798. 28687606 10.1085/jgp.201611734 PMC5560775

[16] Dilly KW, Kurokawa J, Terrenoire C, et al Overexpression of beta2-adrenergic receptors cAMP-dependent protein kinase phosphorylates and modulates slow delayed rectifier potassium channels expressed in murine heart: evidence for receptor/channel co-localization. J Biol Chem. 2004;279:40778–40787. 15272004 10.1074/jbc.M406010200

[17] Li Y, Chen L, Kass RS, et al The A-kinase anchoring protein yotiao facilitates complex formation between adenylyl cyclase type 9 and the IKs potassium channel in heart. J Biol Chem. 2012;287:29815–29824. 22778270 10.1074/jbc.M112.380568 PMC3436180

[18] Kurokawa J, Bankston JR, Kaihara A, et al KCNE variants reveal a critical role of the beta subunit carboxyl terminus in PKA-dependent regulation of the IKs potassium channel. Channels. 2009;3:16–24. 19077539 10.4161/chan.3.1.7387 PMC2773666

[19] Werry D, Eldstrom J, Wang Z, et al Single-channel basis for the slow activation of the repolarizing cardiac potassium current, I(Ks). Proc Natl Acad Sci USA. 2013;110:E996–E1005. 23431135 10.1073/pnas.1214875110 PMC3600500

[20] Eldstrom J, Wang Z, Werry D, et al Microscopic mechanisms for long QT syndrome type 1 revealed by single-channel analysis of I(Ks) with S3 domain mutations in KCNQ1. Heart Rhythm. 2015;12:386–394. 25444851 10.1016/j.hrthm.2014.10.029

[21] Eldstrom J, Xu H, Werry D, et al Mechanistic basis for LQT1 caused by S3 mutations in the KCNQ1 subunit of I(Ks). J Gen Physiol. 2010;135:433–448. 20421371 10.1085/jgp.200910351 PMC2860592

[22] Kurokawa J, Chen L, Kass RS. Requirement of subunit expression for cAMP-mediated regulation of a heart potassium channel. Proc Natl Acad Sci USA. 2003;100:2122–2127. 12566567 10.1073/pnas.0434935100 PMC149969

[23] Li Y, Zaydman MA, Wu D, et al KCNE1 enhances phosphatidylinositol 4,5-bisphosphate (PIP(2)) sensitivity of I(Ks) to modulate channel activity. Proc Natl Acad Sci USA. 2011;108:9095–9100. 21576493 10.1073/pnas.1100872108 PMC3107281

